# Health and House Warming

**Published:** 1900-02-17

**Authors:** 


### Health and House Warming.

Few things are more calculated to diminish the comfort of many households than an increase in the price of fuel; and the sudden setting in of frost, with coal at an approach to forty shillings a ton, is likely to exert a prejudicial influence on the health and welfare of large numbers of comparatively poor people. This is especially the case during the prevalence of influenza, a malady always associated with an elevation of temperature during its active stage, and generally with a corresponding reduction when that stage has passed away. Any additional depression of temperature occasioned by the coldness of the surrounding air is most prejudicial, and on the other hand the preservation of animal heat is of material assistance in enabling the system to resist the invasion of the influenza microbe, or to deal successfully with the invaders if their presence should be displayed by the customary signs. On this particular ground during the present time, and on general grounds at all times, it is probably true that a diminution of domestic warmth is perhaps the most dangerous and most pernicious form which household "economy" can assume; and yet, in the homes of those families who are compelled to keep within the verge of a narrow income, it is usually one of the first to be adopted. It is so much so that it is hardly possible to doubt that a considerable increase in the cost of coal would always bring about a corresponding increase of mortality, at all ages during the existence of an influenza epidemic, and notably among children and the aged even at other times. It becomes the duty of public instructors to call attention to the dangers which may in this way be incurred, and to point out that, with due regard for health and life, domestic expenditure on food and fuel should be among the last forms in which mere saving should be attempted.

Mr. Pickard, M.P., the president of the Miners' Federation of Great Britain, has written a letter to the *Times* officially denying that the increased price of coal is due to any increase of demands on the part of the miners, and showing that such increase as they have actually obtained during the last two years amounts to no more than threepence per ton. In commenting upon this, the *Times* attributes the present prices mainly to a great increase in the activity of all forms of manufacturing industry, and to a consequent increase in the demand for coal, adding that the stimulus thus given to production has already caused the opening of new pits, which, as they come into full working activity, will tend towards the restoration of prices to their former level. This may be true, and we hope it is, but, at the same time, it is difficult entirely to banish Mr. Pickard's suggestion that the increase is attributable to the acts of "some person or persons very deeply interested in the sale of coal." The Coal Exchange affords great facilities for transactions which are more calculated to promote the prosperity of middlemen than the advantage of the public; and we cannot help thinking that in this direction, rather than in any other, may be found the explanation of an enhancement of price which, in all probability, is much in excess of any real enhancement of demand. It has been said that the abolition of the coal duty once levied in the City of London, and applied to purposes of public improvement, instead of being a relief to the dwellers in London was chiefly of advantage to the "trade." A combination, real or suspected, among traders should be met by a similar combination among consumers; and every householder should endeavour, without diminution of domestic warmth, to cut down the use of coal to the lowest possible point. The gas companies will afford great facilities for the substitution of gas for coal both as a warming and a cooking agent; petroleum oil can be conveniently burnt in many forms of stoves; and an admixture of gas coke with coal will afford a fire of great endurance and of excellent warmth. Something might also be done by the great co-operative societies, the Army and Navy and the Civil Service, to assist not only their own members, but also consumers in general. The magnitude of the transactions in which these societies are engaged should enable them to go directly to the pits, and to meet the so-called "merchants" upon terms of equality. To whatever extent they could in this way checkmate the proceedings of monopolists, they would confer benefits upon the public which the public, on its part, would not be slow to recognise.

